# Involvement of FSP1-CoQ_10_-NADH and GSH-GPx-4 pathways in retinal pigment epithelium ferroptosis

**DOI:** 10.1038/s41419-022-04924-4

**Published:** 2022-05-18

**Authors:** Ming Yang, Michelle Grace Tsui, Jessica Kwan Wun Tsang, Rajesh Kumar Goit, Kwok-Ming Yao, Kwok-Fai So, Wai-Ching Lam, Amy Cheuk Yin Lo

**Affiliations:** 1 Department of Ophthalmology, Li Ka Shing Faculty of Medicine, The University of Hong Kong, Hong Kong, China; 2 School of Biomedical Sciences, Li Ka Shing Faculty of Medicine, The University of Hong Kong, Hong Kong, China; 3 State Key Laboratory of Brain and Cognitive Sciences, The University of Hong Kong, Hong Kong, China; 4 GHM Institute of CNS Regeneration, Jinan University, Guangzhou, China

**Keywords:** Apoptosis, Neurodegenerative diseases, Experimental models of disease

## Abstract

Retinal pigment epithelium (RPE) degeneration plays an important role in a group of retinal disorders such as retinal degeneration (RD) and age-related macular degeneration (AMD). The mechanism of RPE cell death is not yet fully elucidated. Ferroptosis, a novel regulated cell death pathway, participates in cancer and several neurodegenerative diseases. Glutathione peroxidase 4 (GPx-4) and ferroptosis suppressor protein 1 (FSP1) have been proposed to be two main regulators of ferroptosis in these diseases; yet, their roles in RPE degeneration remain elusive. Here, we report that both FSP1-CoQ_10_-NADH and GSH-GPx-4 pathways inhibit retinal ferroptosis in sodium iodate (SIO)-induced retinal degeneration pathologies in human primary RPE cells (HRPEpiC), ARPE-19 cell line, and mice. GSH-GPx-4 signaling was compromised after a toxic injury caused by SIO, which was aggravated by silencing GPx-4, and ferroptosis inhibitors robustly protected RPE cells from the challenge. Interestingly, while inhibition of FSP1 caused RPE cell death, which was aggravated by SIO exposure, overexpression of FSP1 effectively protected RPE cells from SIO-induced injury, accompanied by a significant down-regulation of CoQ_10_/NADH and lipid peroxidation. Most importantly, in vivo results showed that Ferrostatin-1 not only remarkably alleviated SIO-induced RPE cell loss, photoreceptor death, and retinal dysfunction but also significantly ameliorated the compromised GSH-GPx-4 and FSP1-CoQ_10_-NADH signaling in RPE cells isolated from SIO-induced RPE degeneration. These data describe a distinct role for ferroptosis in controlling RPE cell death in vitro and in vivo and may provide a new avenue for identifying treatment targets for RPE degeneration.

## Introduction

Retinal pigment epithelium (RPE) is a single layer of pigment cells that lies between the neural retina and choroid. It consists of ~5 million hexagonal RPE cells attaching to its basal lamina (Bruch’s membrane) [1]. RPE degeneration plays an important role in a range of retinal disorders such as retinal degeneration and age-related macular degeneration [2]. Oxidative stress is considered a key mechanism for RPE degeneration [3, 4]. The imbalance between the oxidation and antioxidation systems in the retina increases the production of highly reactive molecular oxygen free radicals (ROS) and nitrogen free radicals, which in turn leads to RPE degeneration [4–6].

The process of cell death has been considered unregulated until the concept of programmed cell death (PCD) was proposed [7]. Nowadays, cell death is classified into two categories: accidental cell death and regulated cell death (RCD) [8]. Despite previous studies on retina cell damage associated with oxidative stress [4, 9, 10], retina cell death mechanisms are still not fully understood. Many agents have been used to induce retinal cell death, one of which is sodium iodate (SIO), an oxidizer that is toxic to RPE and neuroretina, making it a suitable model for RPE degeneration [11–13]. There has been one report on necroptosis-associated RPE injury after SIO treatment; [14] yet, the cell death mechanism of SIO-mediated RPE damage is still not fully elucidated.

Ferroptosis was first proposed in 2012 by Dixon et al. [15]. It is an iron-dependent and non-apoptotic cell death, with distinctive features including iron overload, lipid peroxidation, and mitochondrial destruction [16]. Current research has classified ferroptosis into several pathways: (1) depletion of glutathione (GSH), with a decreased activity of GPx-4 (GSH-GPx-4 pathway) [15, 16]; (2) GTP cyclohydrolase-1 (GCH1) and tetrahydrobiopterin/dihydrobiopterin (BH_4_/BH_2_). BH_4_ and BH_2_ are metabolic derivatives of GCH1, which contribute to lipid remodeling and ferroptosis suppression [17]; and (3) decreased activity of ferroptosis suppressor protein 1 (FSP1), accompanied by the exhaustion of CoQ_10_ (FSP1-CoQ_10_-NADH pathway) [18, 19].

Besides the discovery of hydrogen-peroxide-associated RPE ferroptosis [20], little is known about SIO-induced ferroptosis in primary human RPE cells and in vivo. This study aimed to elucidate the role of ferroptosis in SIO-induced RPE degeneration in vitro and in vivo models using gain of function and loss of function strategies. The characterization of ferroptosis involvement in RPE degeneration pathogenesis will aid the search for treatment targets for RPE degeneration.

## Results

### SIO-induced RPE death mimics RPE degeneration in vitro and in vivo

**Fig. 1:**
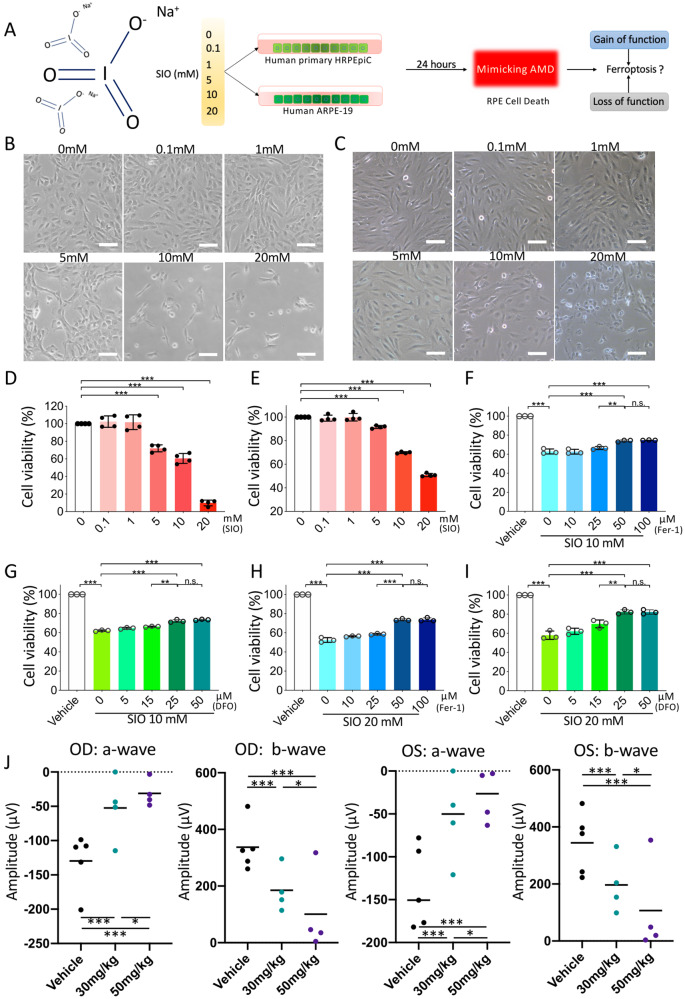
The establishment of SIO-induced RPE degeneration in vitro and in vivo. **A** A flowchart showing the in vitro study design. SIO at different concentration or PBS was added to human primary HRPEpiC cells and ARPE-19 cells for 24 h to establish RPE degeneration models in vitro. SIO sodium iodate, RPE retinal pigment epithelium, HRPEpiC human retinal pigment epithelial cells, ARPE-19 adult retinal pigment epithelial cells, AMD age-related macular degeneration, RD RPE degeneration, PBS phosphate-buffered saline. **B** Primary HRPEpiC morphology and **C** ARPE-19 morphology upon exposure to different concentrations of SIO under phase-contrast microscopy (scale bar = 100 µm). **D** CCK-8 cell viability assay results of primary HRPEpiC upon exposure to different concentrations of SIO. CCK-8 Cell Counting Kit-8. *n* = 4 (independent experiments). **E** CCK-8 cell viability assay results of ARPE-19 upon exposure to different concentrations of SIO. *n* = 3 (independent experiments). **F** CCK-8 cell viability assay results of the effect of Fer-1 pre-treatment on 10 mM SIO-challenged primary HRPEpiC cells. Different concentrations of Fer-1 were prepared in DMSO, followed by SIO treatment. The vehicle is the control for Fer-1. Fer-1 Ferrostatin-1. *n* = 3. **G** CCK-8 cell viability assay results of the effect of DFO pre-treatment on 10 mM SIO-challenged primary HRPEpiC cells. DFO was dissolved in 1xPBS first and used for later treatment. The vehicle is the control for DFO. DFO deferoxamine. *n* = 3. **H** CCK-8 cell viability assay results of the effect of Fer-1 pre-treatment on 20 mM SIO-challenged ARPE-19 cells. Fer-1 is dissolved in DMSO first and used for later treatment. The vehicle is the control for Fer-1. *n* = 3 (independent experiments). **I** CCK-8 cell viability assay results of the effect of DFO pre-treatment on 20 mM SIO-challenged ARPE-19 cells. Different concentrations of DFO were prepared in 1×PBS, followed by SIO treatment. The vehicle is the control for DFO. *n* = 3. **J** Mice were subjected to two dosages of SIO and ERG test was performed on the 3rd day after intraperitoneal injection. ERG amplitude results of mice in two dosages of SIO on the 3rd day after injection. *n* = 4–5 (independent experiments). *p < 0.05, **p < 0.01, ***p < 0.001.

To determine the optimal SIO concentration for inducing RPE cell death, primary HRPEpiC and ARPE-19 cells were exposed to SIO at 0, 0.1, 1, 5, 10, 20 mM for 24 h, respectively (Fig. 1A). Primary HRPEpiC cells showed significant morphological changes after the 10 mM SIO challenge, displaying ruptured cell membrane and cell debris as well as a significant decrease in cell density (Fig. 1B). Similar results were obtained in ARPE-19 cells (Fig. 1C). Cell Counting Kit-8 (CCK-8) assay showed that SIO decreased primary HRPEpiC cell viability in a dose-dependent manner, with only around 15% of cells surviving at 20 mM SIO (*p* < 0.001, Fig. 1D). In contrast, ARPE-19 cells were more resistant; cell viability was around 50% at 20 mM SIO (Fig. 1E). We selected the dosages (10 and 20 mM) that cause around 50% cell death in primary HRPEpiC and ARPE-19 cells, respectively for further studies.

The doses of ferrostatin-1 (Fer-1) and deferoxamine (DFO) (two inhibitors of ferroptosis) used were also optimized in SIO-treated primary HRPEpiC and ARPE-19 cells. Primary HRPEpiC cell viability increased with Fer-1 or DFO treatment, reaching a maximum of 77.45% for Fer-1 (50 µM) and 73.92% for DFO (25 µM) (*p* < 0.001, Fig. 1F, G). Similar results were obtained for ARPE-19 (Fig. 1H, I). Therefore, Fer-1 (50 µM) and DFO (25 µM) were selected for subsequent studies.

Intraperitoneal SIO injection (30 or 50 mg/kg) in mice was performed to establish the RPE degeneration in vivo model as described previously [21]. Electroretinogram (ERG) is used to test the electrical activity and responsiveness of the retina upon light stimulation [22]. It represents the physiological health of the retina. The waveforms generated are separated into two components: a-wave followed by b-wave. The a-wave is generated by the photoreceptor while b-wave reflects the response from the bipolar cells and other layers of the outer retina. ERG was therefore measured as reported [23–26] to reflect retinal function. ERG showed that there were significant decreases in both a- and b-wave amplitudes 3 days after SIO administration, indicating the successful establishment of the model (Fig. 1J, *p* < 0.001). In this study, we, therefore, selected a single intraperitoneal injection of SIO (30 mg/kg) for 3 days as the in vivo model for RPE degeneration.

### Ferroptosis regulates SIO-induced cell death in RPE cells by GSH-GPx-4 pathway

**Fig. 2:**
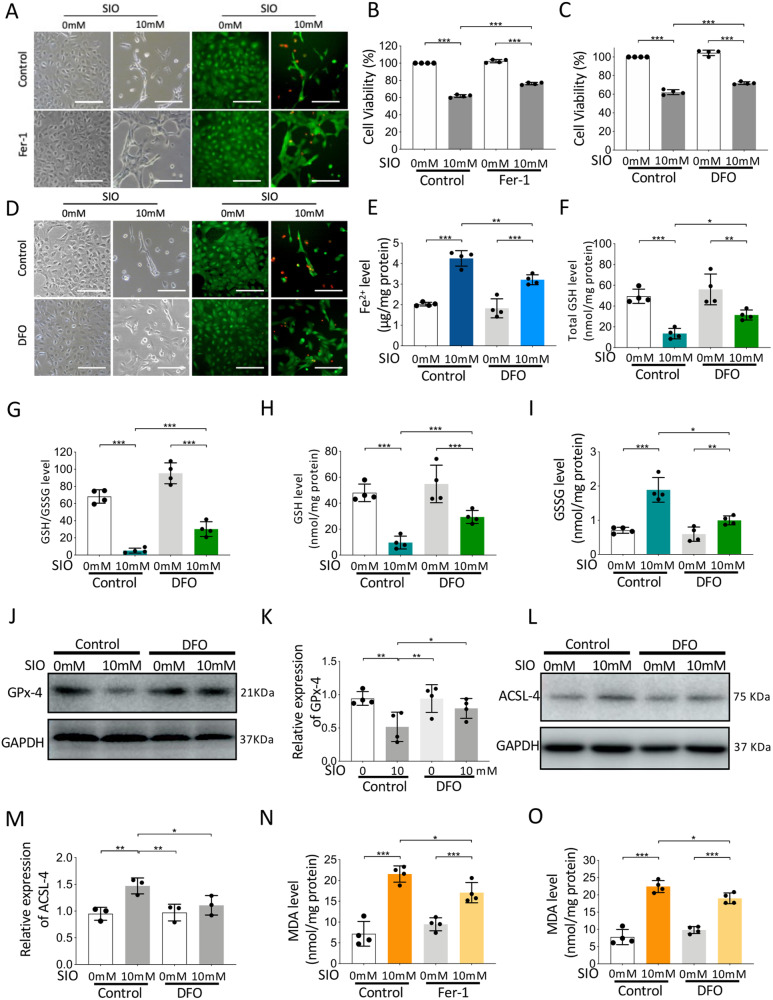
Ferroptosis regulates SIO-induced human primary HRPEpiC cell death. Fer-1 (50 µM) or its vehicle (0.14% DMSO) was added to human primary HRPEpiC cells for 3 h before control (PBS) or SIO (10 mM) treatment. DFO (25 µM) or its vehicle (PBS) was added to human primary HREpiC cells 3 h before control (PBS) or SIO (10 mM) treatment. **A** Phase-contrast and fluorescence microscope images of cell morphology and Live/dead cell staining of SIO-exposed human primary HRPEpiC cells with/without Fer-1 pre-treatment are shown. Scale bar = 100 µm. **B** CCK-8 cell viability assay results of the effect of Fer-1 on 10 mM SIO-induced human primary HRPEpiC cell death. *n* = 4 (independent experiments). **C** CCK-8 cell viability assay results of the effect of DFO on 10 mM SIO-induced human primary HRPEpiC cell death. *n* = 4 (independent experiments). **D** Phase-contrast and fluorescence microscope images of cell morphology and Live/dead cell staining of SIO-exposed human primary HRPEpiC cells with/without DFO pre-treatment are shown. Scale bar = 100 µm. *n* = 4 (independent experiments). **E** Labile iron levels in SIO-exposed human primary HRPEpiC cells with/without DFO pre-treatment. *n* = 4 independent experiments. **F** Total GSH level of SIO-exposed human primary HRPEpiC cells with/without DFO pre-treatment. *n* = 4 (independent experiments). **G** GSSG level of SIO-exposed human primary HRPEpiC cells with/without DFO pre-treatment. *n* = 4 (independent experiments). **H** GSH level of SIO-exposed human primary HRPEpiC cells with/without DFO pre-treatment. *n* = 4 (independent experiments). **I** GSH/GSSG ratio of SIO-exposed human primary HRPEpiC cells with/without DFO pre-treatment. *n* = 4 (independent experiments). **J** The expression of GPx-4 of SIO-exposed human primary HRPEpiC cells with/without DFO pre-treatment showed by Western blot. **K** Quantitative analysis of the expression of GPx-4. *n* = 4 (independent experiments). **L** The expression of ACSL-4 of SIO-exposed human primary HRPEpiC cells with/without DFO pre-treatment showed by Western blot. **M** Quantitative analysis of the expression of ACSL-4. *n* = 3 (independent experiments). **N** MDA level of SIO-exposed human primary HRPEpiC cells with/without Fer-1 pre-treatment. *n* = 4 (independent experiments). **O** MDA level of SIO-exposed human primary HRPEpiC cells with/without DFO pre-treatment. *n* = 4 (independent experiments). *p < 0.05, **p < 0.01, ***p < 0.001.

To investigate the role of ferroptosis in primary HRPEpiC cell death induced by SIO, Fer-1, and DFO, two classical ferroptosis inhibitors were used. Initially, 10 mM SIO significantly induced cell damage and reduced cell viability (Fig. 2A, B, *p* < 0.001). LIVE/DEAD cell imaging assay also showed increased red signals, indicating cell death with a decreased live-cell green signal (Fig. 2A). However, cells pre-treated with 50 μM Fer-1 for 3 h showed less damage with higher cell viability and fewer red signals, indicating that Fer-1 rescued SIO-induced primary HRPEpiC cell death (Fig. 2A). Meanwhile, primary HRPEpiC cells after 25 μM DFO pre-treatment displayed decreased cell death and morphological changes upon SIO stress (Fig. 2C, D). These results suggested that ferroptosis is involved in SIO-induced primary HRPEpiC cell death.

To further understand how ferroptosis mediated primary HRPEpiC cell death upon SIO exposure, intracellular iron level, glutathione metabolism, GPx-4 expression, and lipid peroxidation were assayed. As shown in Fig. 2E, SIO significantly increased intracellular labile iron level, which was reduced with 3 h of iron chelator DFO pre-treatment. In addition, while intracellular total GSH (Fig. 2F, *p* < 0.001), GSH/GSSG (Fig. 2G, *p* < 0.001), and GSH (Fig. 2H, *p* < 0.001) showed a remarkable decrease upon SIO exposure, GSSG level was significantly increased (Fig. 2I), indicating exhaustion of glutathione metabolism. However, DFO pre-treatment alleviated this change, suggesting the involvement of GSH signaling in SIO-induced primary HRPEpiC ferroptosis.

Next, the expression of GPx-4, the key enzyme in glutathione metabolism and ferroptosis [27] was measured. A significant decrease of GPx-4 expression upon SIO stress was observed, and this was mitigated by pre-treatment with DFO (Fig. 2J, K, *p* < 0.001). We further assayed the level of long-chain-fatty-acid-CoA ligase 4 (ACSL-4), one of the key enzymes contributing to lipid peroxidation and malondialdehyde (MDA), a marker of lipid peroxidation. The results showed that SIO significantly elevated both ACSL-4 expression (Fig. 2L, M, *p* < 0.001) and MDA level (Fig. 2N, *p* < 0.001), but these were attenuated by Fer-1 pre-treatment (*p* < 0.001). To further confirm this, we also tested DFO, another ferroptosis inhibitor that elicited a similar decrease in MDA level (Fig. 2O, *p* < 0.001). As GPx-4 is the key regulator of the classical ferroptosis pathway, we also adopted a loss-of-function strategy, i.e., GPx-4 siRNA transfection assay to further confirm the role of ferroptosis in SIO-induced cell death. SIO significantly increased the immunofluorescence intensity of 4-Hydroxynonenal (4-HNE) and this up-regulation was stronger in primary HRPEpiC cells transfected with GPx-4 siRNA (Fig. S1A, B, *p* < 0.001). In addition, transmission electron microscopy (TEM) showed typical ferroptosis-related morphological features in SIO-induced primary HRPEpiC cells; decreased mitochondria size with increased membrane density were observed. These mitochondrial morphological changes were more apparent in GPx-4 knockdown cells compared with control (Fig. S1C). These data suggested that ferroptosis in primary HRPEpiC cells induced by SIO is mediated through the GSH-GPx-4 pathway.

Using the same pharmacological strategies, we also studied the role of SIO-induced ferroptosis in ARPE-19 cells. Consistently, SIO drastically decreased ARPE-19 cell viability but this was significantly ameliorated by 50 μM Fer-1 (Fig. S2A, B) or 25 μM DFO pre-treatment for 3 h (Fig. S2C, E), suggesting that Fer-1 and DFO protect against SIO-induced ARPE-19 cell death. Cell morphology, cell viability, intracellular iron level, glutathione metabolism, GPx-4 expression, and lipid peroxidation were further assayed in SIO-challenged ARPE-19 cells. The results were similar to those observed in primary HRPEpiC cells (Fig. S2A–K). Most importantly, ARPE-19 cells transfected with GPx-4 siRNA showed more significant morphological damage and reduced cell viability. There was a further increase in red signals in the LIVE/DEAD cell imaging assay, indicating that enhancement of ferroptotic activity aggravated SIO-induced cell death (Fig. S2O, R). In addition, SIO significantly increased the immunofluorescence intensity of 4-HNE and this up-regulation was stronger in cells transfected with GPx-4 siRNA (Fig. S2Q, *p* < 0.001). Furthermore, GPx-4 siRNA transfected cells showed more significantly damaged cell morphology, and reduced cell viability with further increased red signals, indicating that enhancing the activity of ferroptosis by reduction of GPx-4 levels aggravated SIO-induced cell death (Fig. S2R). Moreover, TEM revealed dense and shrunken mitochondria in SIO-exposed ARPE-19 cells, which were aggravated upon GPx-4 knockdown (Fig. S2S). These data suggest that GSH-GPx-4 pathway regulates ferroptosis in SIO-induced ARPE-19 cell damage.

### Fer-1 significantly improved retinal function in the SIO-induced RPE degeneration model in vivo

**Fig. 3:**
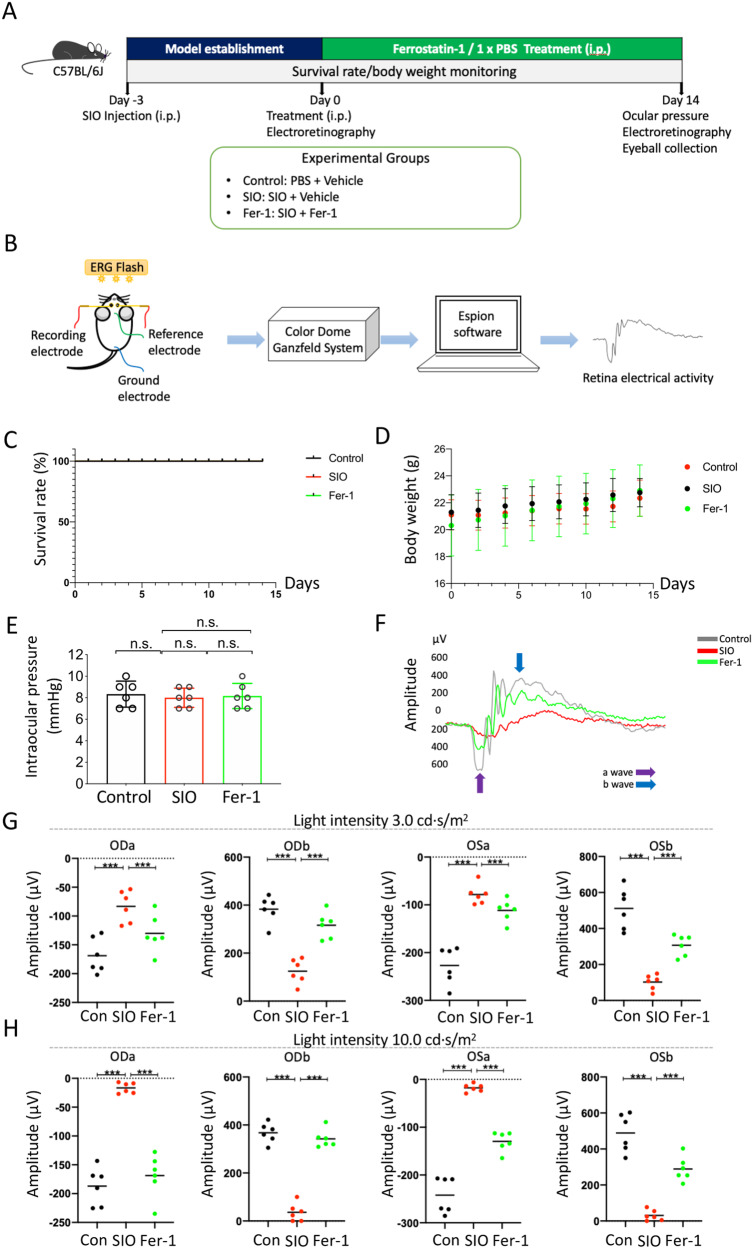
A study design for assessment of the effect of Fer-1 on SIO-induced RPE degeneration in vivo. **A** Timeline of the study. On day 3, mice received a single intraperitoneal injection of SIO (30 mg/kg). On day 0, RPE degeneration phenotype was established at which daily treatment with Ferrostatin-1 (Fer-1) commenced and lasted for 14 days. The effect of Fer-1 in SIO-challenged mice was evaluated on day 14. **B** A schematic diagram showing ERG experimental procedures in mice. **C** Survival rate monitoring of mice receiving SIO and/or Fer-1 injections. **D** Body weight monitoring of mice receiving vehicle, SIO and/or Fer-1 injections. **E** Intraocular pressure in mouse eyes after SIO and/or Fer-1 treatment on day 14. *n* = 6 (biological replicates). n.s. no statistical significance. **F** Representative ERG waveform of mouse retina after SIO and/or Fer-1 treatment on day 14. **G** a- and b-wave amplitudes measured at 3 cd s/m^2^ in mice receiving vehicle, SIO and/or Fer-1 injections. ODa a-wave in the right eye, ODb b-wave in the right eye, OSa a-wave in the left eye, OSb b-wave in the left eye. *n* = 6 (biological replicates). **H** a- and b-wave amplitudes measured at 10 cd s/m^2^ in mice receiving vehicle, SIO and/or Fer-1 injection. *n* = 6 (biological replicates). *p < 0.05, **p < 0.01, ***p < 0.001.

To investigate the effect of Fer-1 on SIO-induced RPE cell death in vivo, we monitored survival rate, body weight, intraocular pressure, and retinal function in SIO-injected mice with/without Fer-1 treatment (Fig. 3A, B). As shown in Fig. 3C, there was no change in survival rate among SIO-challenged, Fer-1-treated, and vehicle controls throughout the 14 days of treatment. There was a slight increase in body weight in all 3 groups (Fig. 3D). No difference in intraocular pressure (IOP) could be observed among the three groups (Fig. 3E). However, SIO significantly decreased the amplitudes of ERG a-wave and b-wave in both eyes when tested at 3 and 10 cd s/m^2^, which were subsequently rescued after daily injection of Fer-1 for 14 days (Fig. 3F–H, *p* < 0.001). These data not only showed the protective effect of Fer-1 on SIO-induced retinal function loss in the RPE degeneration mouse model but also suggested the potential therapeutic role of ferroptosis in RPE degeneration.

### Ferroptosis is involved in SIO-induced RPE degeneration in vivo

**Fig. 4:**
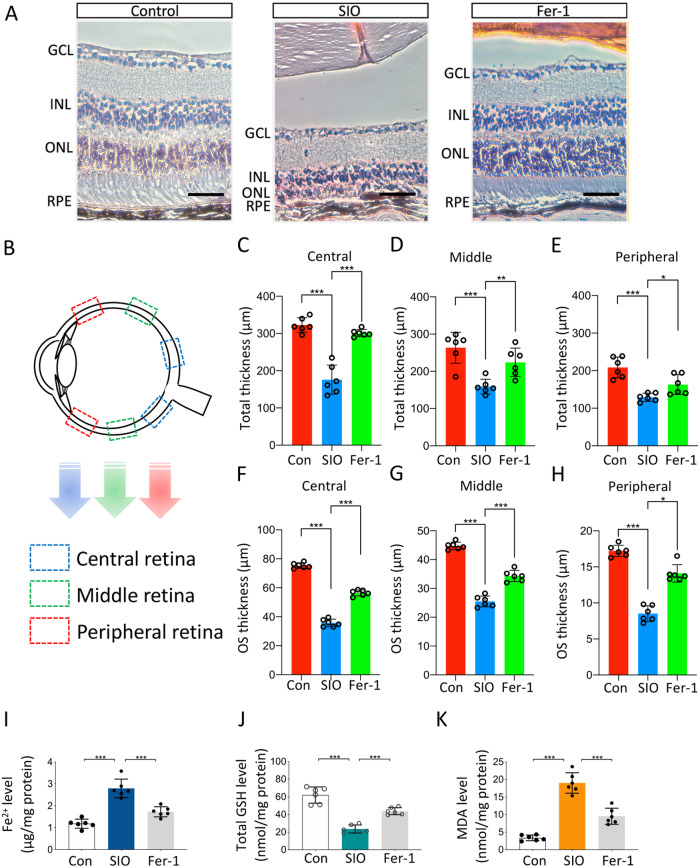
Ferroptosis regulates SIO-induced RPE degeneration in vivo. **A** H&E-stained retinal sections from mice receiving vehicle, SIO, and/or Fer-1 administration. Scale bar = 200 µm. **B** A schematic diagram showing the location of areas on retinal sections used for total retinal thickness and outer segment thickness measurement. **C** Quantitative analysis of total retinal thickness (central). *n* = 6 (biological replicates). **D** Quantitative analysis of total retinal thickness (middle). *n* = 6 biological replicates. **E** Quantitative analysis of total retinal thickness (peripheral). *n* = 6 (biological replicates). **F** Quantitative analysis of outer photoreceptor thickness (central). *n* = 6 (biological replicates). **G** Quantitative analysis of photoreceptor outer segment (OS) thickness (middle). *n* = 6 (biological replicates). **H** Quantitative analysis of photoreceptor outer segment (OS) (peripheral). *n* = 6 (biological replicates). **I** Labile iron level in RPE after SIO challenge with/without Fer-1 pre-treatment. *n* = 6 (biological replicates). **J** Total GSH level of RPE in SIO-induced RPE degeneration with/without Fer-1 pre-treatment. *n* = 6 (biological replicates). **K** MDA level of RPE in SIO-induced RPE degeneration with/without Fer-1 pre-treatment. *n* = 6 (biological replicates). *p < 0.05, **p < 0.01, ***p < 0.001.

To confirm the involvement of ferroptosis in the SIO-induced RPE degeneration mouse model, mouse eyeballs were collected, sectioned, and analyzed. Cytoarchitectural analysis on hematoxylin-and-eosin (H&E)-stained retinal sections showed that SIO significantly decreased retinal thickness and caused massive RPE cell loss (Fig. 4A). However, the Fer-1 treatment rescued the retina; it not only restored retinal thickness and rescued RPE cells (Fig. 4C–E, *p* < 0.001) but also protected the photoreceptor outer segments (Fig. 4F–H, *p* < 0.001). Meanwhile, there was also a significant increase in intracellular iron in isolated RPE cells from mouse retina after the SIO challenge, suggesting that labile iron was increased in vivo; yet, the iron overload in RPE was alleviated after Fer-1 treatment for 14 days (Fig. 4I, *p* < 0.001). Moreover, the GSH level in RPE was significantly decreased after the SIO challenge; but Fer-1 treatment again rescued the pathological change and restored the RPE GSH level (Fig. 4J, *p* < 0.001). To test for the presence of lipid peroxidation, we measured MDA levels in RPE. As shown in Fig. 4K, the SIO challenge induced an apparent increase in MDA level. However, this was significantly suppressed by 14 days of Fer-1 treatment. Taken together, these data supported the involvement of ferroptosis in the SIO-induced RPE degeneration model in vivo.

### FSP1-CoQ_10_-NADH may also be involved in SIO-induced ferroptosis

**Fig. 5:**
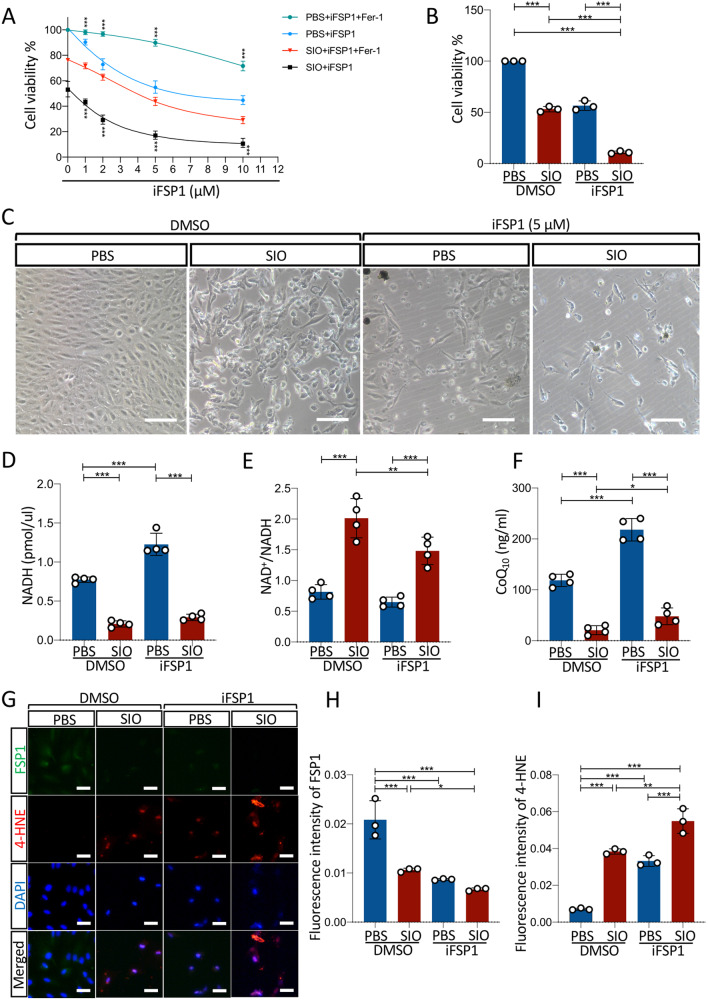
Inhibition of FSP1 aggravated SIO-induced ARPE-19 cell death through FSP1-CoQ_10_-NADH pathway. **A** CCK-8 cell viability assay results of the effect of iFSP1 on 20 mM SIO-induced ARPE-19 cell death. *n* = 3 (independent experiments). **B** CCK-8 cell viability assay results of the effect of 5 µM iFSP1 on 20 mM SIO-induced ARPE-19 cell death. *n* = 3 (independent experiments). **C** Phase-contrast microscope images of cell morphology of SIO-exposed ARPE-19 cells with/without iFSP1 treatment are shown. Scale bar = 100 µm. **D** NADH level of SIO-induced ARPE-19 cell death with/without iFSP1 treatment. *n* = 4 (independent experiments). **E** NAD^+^/NADH ratio of SIO-induced ARPE-19 cell death with/without iFSP1 treatment. *n* = 4 (independent experiments). **F** CoQ_10_ level of ARPE-19 in SIO-induced RPE degeneration with/without iFSP1 treatment. *n* = 4 (independent experiments). **G** FSP1 and 4-HNE immunofluorescence signals in SIO-exposed ARPE-19 cells with or without FSP1 DNA transfection ARPE-19 Scale bar = 50 µm. **H** Quantitative analysis of the fluorescent intensity of FSP1. *n* = 3 (independent experiments). **I** Quantitative analysis of the fluorescent intensity of 4-HNE. *n* = 3 (independent experiments). *p < 0.05, **p < 0.01, ***p < 0.001.

To evaluate the role of FSP1 on SIO-induced ferroptosis, we performed pharmacological and gain-of-function experiments on ARPE-19 cells. As shown in Fig. 5A, a dose-dependent drop in cell viability was observed in ARPE-19 cells treated with iFSP1 (blue line). Fer-1 was able to reduce the decreased cell viability at all iFSP1 dosages tested (green line), suggesting that iFSP1 induces cell death via ferroptosis that can be blocked by Fer-1. SIO treatment on cells incubated with iFSP1 further decreased cell viability (black line). Most importantly, Fer-1 could again significantly reduce the decrease in cell viability caused by both SIO and iFSP1 administration in a dose-dependent manner (red line).

These findings are consistent with other studies showing the effect of iFSP1 is compromised by the treatment addition of ferroptosis inhibitors such as Fer-1 [28] and liproxstatin-1 [18]. Control normalization for SIO and iFSP1 was also performed to eliminate the possibility of a vehicle effect (Fig. S3A). As expected, no significant cell viability change was observed when there was an increased concentration of DMSO present in the 20 mM SIO-treated cells. Moreover, the effect of iFSP1 on SIO dose-response was tested. As shown in Fig. S3B–H, the slopes of SIO dose-response with 1 µM iFSP1 (*n* = 3) were not statistically different from those in the vehicle control group (*p* = 0.525, *n* = 3, Levene’s test), suggesting that the dose-response of ARPE-19 cells to SIO challenge in the presence of 1 µM iFSP1 was similar to that with vehicle treatment and iFSP1 caused no sensitization of SIO dose-response. To further confirm the role of FSP1, we chose iFSP1 at a dose of 5 μM, which induced the most significant effect on cell death for further studies. SIO administration significantly decreased cell viability with decreased cell density (Fig. 5B) and noticeable morphological changes (Fig. 5C) compared with the control group. iFSP1 further aggravated these effects, suggesting that FSP1 is required in SIO-induced ARPE-19 ferroptosis. In addition, intracellular NADH level was significantly decreased by SIO treatment but it was increased by iFSP1 administration (Fig. 5D). In contrast, SIO significantly elevated NAD^+^/NADH, which was ameliorated with iFSP1 treatment (Fig. 5E), suggesting that FSP1 might consume NADH in ARPE-19 cells. CoQ_10_ ELISA assay (Fig. 5F) also showed similar results as in the NADH assay. Furthermore, SIO not only significantly decreased the expression of FSP1 but also aggravated the increased fluorescence signal of 4-HNE induced by iFSP1, suggesting intensified lipid peroxidation activity in ARPE-19 cells (Fig. 5G, I).

**Fig. 6:**
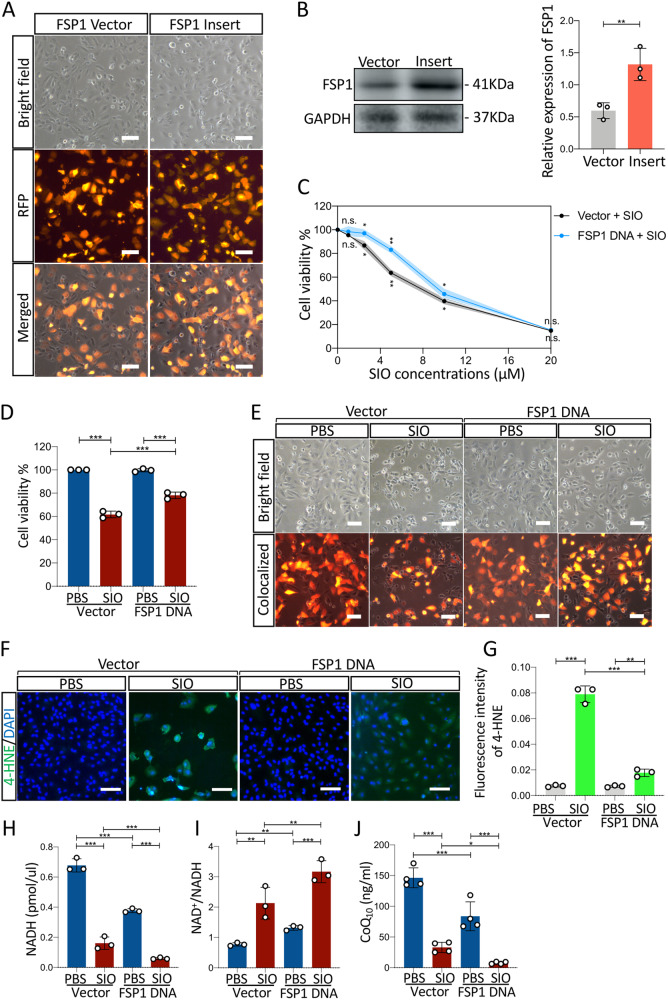
Overexpression of FSP1 alleviated SIO-induced ARPE-19 cell death through FSP1-CoQ_10_-NADH pathway. **A** Phase-contrast microscope images of cell morphology of FSP1 DNA vector and insert transfected ARPE-19 cells. Scale bar = 100 µm. RFP red fluorescence protein, indicating successfully transfected cells. **B** Western blot assay of the protein expression level of FSP1 in FSP1 DNA vector and insert transfected ARPE-19 cells. *n* = 3 (independent experiments). **C** CCK-8 cell viability assay results of the effect of FSP1 DNA transfection on cell viability of ARPE-19 exposed at different concentrations of SIO. *n* = 3 (independent experiments). **D** CCK-8 cell viability assay results of the effect of FSP1 DNA transfection on cell viability of ARPE-19 exposed with or without 5 μM of SIO. *n* = 3 (independent experiments). **E** Phase-contrast microscope images of cell morphology of FSP1 DNA vector and insert transfected ARPE-19 cells with/without 5 μM SIO. **F** 4-HNE immunofluorescence signal in SIO-exposed ARPE-19 cells with/without FSP1 DNA transfection. Scale bar = 100 µm. **G** Quantitative analysis of 4-HNE immunofluorescence signal in SIO-exposed ARPE-19 cells with/without FSP1 DNA transfection. *n* = 3 (independent experiments). **H** NADH level of FSP1 DNA or vector-transfected ARPE-19 cells with/without 5 μM SIO treatment. *n* = 3 (independent experiments). **I** NAD^+^/NADH ratio of FSP1 DNA or vector-transfected ARPE-19 cells with/without 5 μM SIO treatment. *n* = 3 (independent experiments). **J** CoQ_10_ level of FSP1 DNA or vector-transfected ARPE-19 cells with/without 5 μM of SIO treatment. *n* = 4 (independent experiments). *p < 0.05, **p < 0.01, ***p < 0.001.

**Fig. 7:**
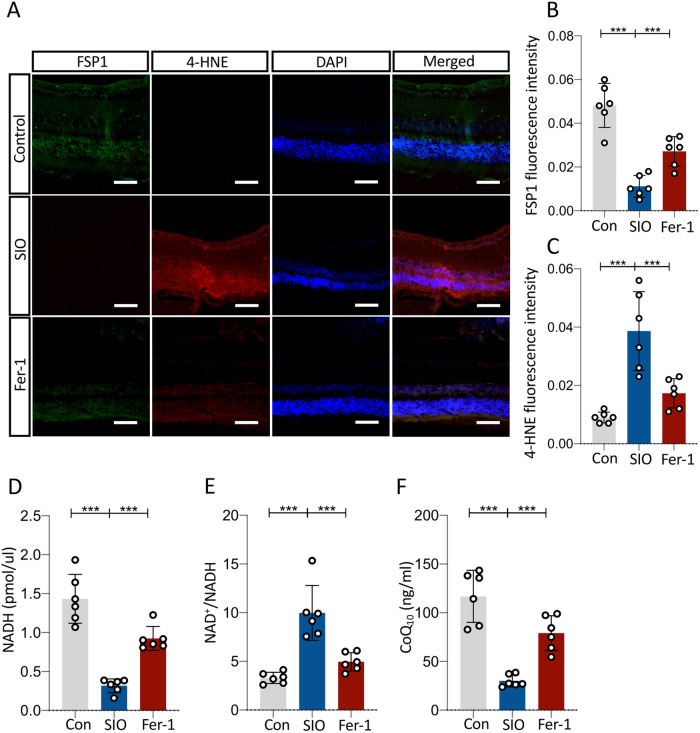
FSP1-CoQ_10_-NADH pathway is involved in SIO-induced retinal pathology resembling age-related macular degeneration in vivo. **A** 4-HNE/FSP1 immunofluorescence in retinal sections of mice receiving vehicle, SIO and/or Fer-1 injections. Scale bar = 200 µm. **B** Quantitative analysis of fluorescent intensity of FSP1. *n* = 6 (biological replicates). **C** Quantitative analysis of fluorescent intensity of 4-HNE. *n* = 6 biological replicates. **D** NADH level of SIO-induced retinal pathology resembling age-related macular degeneration with/without Fer-1 treatment. *n* = 6 (biological replicates). **E** NAD^+^/NADH ratio of SIO-induced retinal pathology resembling age-related macular degeneration with/without Fer-1 treatment. *n* = 6 (biological replicates). **F** CoQ_10_ level of SIO-induced retinal pathology resembling age-related macular degeneration with/without Fer-1 treatment. *n* = 6 (biological replicates). *p < 0.05, **p < 0.01, ***p < 0.001.

**Fig. 8:**
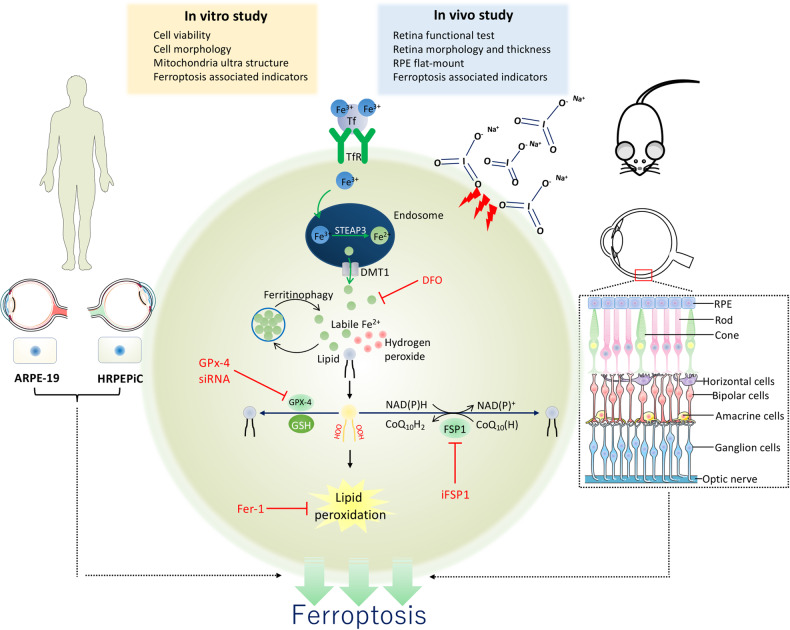
A schematic diagram showing ferroptosis controls the fate of RPE cell death in SIO-induced RPE degeneration in vitro and in vivo. Tf Transferrin, TfR Transferrin receptor, Fe^3+^ Ferric iron, Fe^2+^ Ferrous, STEAP3 Six-Transmembrane Epithelial Antigen Of Prostate 3, DMT1 Lipid, ROS Lipid reactive oxygen species, GPx-4 Glutathione peroxidase-4, GSH Glutathione, DFO Deferoxamine, Fer-1 Ferrostatin-1, OOH Hydroperoxides. The exposure of SIO increased labile iron, which interacts with lipid and hydrogen-peroxide thereby causing Fenton reaction and lipid peroxidation. GPx-4 relies on GSH to eliminate lipid ROS. DFO and Fer-1, two ferroptosis inhibitors, rescue RPE cell death induced by SIO. In contrast, GPx-4 siRNA aggravates SIO-induced RPE cell death. (Parts of the figure were drawn using pictures from Servier Medical Art (http://smart.servier.com/) (accessed on 28 June 2021), licensed under a Creative Commons Attribution 3.0 Unported License (https://creativecommons.org/licenses/by/3.0/) (accessed on 28 June 2021)).

To further confirm the role of FSP1 in SIO-induced ARPE-19 cell death, overexpression of FSP1 was performed. ARPE-19 cells were transfected with FSP1 DNA plasmid or vector control DNA for 48 h and subjected to SIO at different concentrations 0, 1, 2.5, 5, 10, 20 μM for 6 h. As shown in Fig. 6A, the red fluorescence protein (RFP) signal indicated a successful transfection of cells by plasmids carrying both RFP and FSP1 DNA. In total, 70–80% of cells are expressing FSP1. A significantly increased FSP1 protein expression could also be observed in the transfected cells (Fig. 6B). As the concentration of SIO increases, cells transfected with vector DNA showed a sharp decrease in cell viability while FSP1-transfected cells exhibited a delayed decrease (Fig. 6C), suggesting a protective effect of FSP1 protein on SIO-challenged cells. The most significant difference in cell viability between vector + SIO group and FSP1 DNA + SIO group was observed when SIO concentration reached 5 μM (Fig. 6C). Therefore, this dosage was chosen in subsequent in vitro studies. Consistently, 5 μM SIO significantly decreased the viability of ARPE-19 cells transfected with vector control DNA (Fig. 6D) with noticeable cell morphological changes (Fig. 6E). However, this was ameliorated in ARPE-19 cells transfected with FSP1 DNA (Fig. 6D) that displayed less alteration of cell shape (Fig. 6E). 4-HNE staining also showed that SIO-induced lipid peroxidation was markedly mitigated by FSP1 overexpression (Fig. 6F, G). In addition, while intracellular NADH level was significantly decreased in SIO-treated ARPE-19 cells transfected with vector control DNA, the decrease was more significant in FSP1-transfected ARPE-19 cells, suggesting that FSP1 might consume NADH in ARPE-19 cells (Fig. 6H). To further confirm this finding, NAD^+^/NADH assays were performed. SIO significantly increased the ratio of NAD^+^/NADH in ARPE-19 cells transfected with vector control DNA (Fig. 6I), and this was more evident in FSP1-transfected ARPE-19 cells, suggesting NADH consumption and subsequent generation of its metabolic product (NAD^+^). Moreover, intracellular CoQ_10_ was measured by ELISA assay. There was a significant decrease in SIO-treated ARPE-19 cells transfected with vector control DNA, and this was even more significant in FSP1-transfected ARPE-19 (Fig. 6J), which was consistent with the NADH result (Fig. 6H). In vivo data also showed that SIO administration significantly decreased the immunofluorescence intensity of FSP1 in different layers of the retina, suggesting impaired ferroptosis-defending ability (Fig. 7A, B). In contrast, SIO increased the fluorescence intensity of 4-HNE, which was widely distributed in various layers of the retina, indicating the presence of overwhelming retinal lipid peroxidation (Fig. 7A, C). In addition, SIO also significantly decreased the levels of NADH (Fig. 7D), and CoQ_10_ (ubiquinone) (Fig. 7F), but increased NAD+/NADH (Fig. 7E), which are downstream markers of the FSP1-CoQ_10_-NADH pathway in ferroptosis. SIO also induced the loss of ZO-1 immunostained cells and increased 4-HNE immunofluorescence, indicating damage to tight junctions and RPE cells (Fig. S4A–C, *p* < 0.001), which is consistent with a recent study [29]. However, the destruction was alleviated by 14 days’ Fer-1 treatment (Fig. S4A–C, *p* < 0.001). These results further supported the involvement of ferroptosis in vivo. Overall, the evidence presented indicated that the FSP1-CoQ_10_-NADH pathway is involved in SIO-induced RPE degeneration and may play an important role in SIO-induced ferroptosis (Fig. 8).

## Discussion

We previously showed that amyloid-β_1-40_ oligomers-induced pyroptosis in APRE-19 cells [30] and proposed that novel RCD may contribute to a new concept on the pathogenesis of retinal diseases [31]. In this study, we clarified the likely role of ferroptosis associated with both GSH-GPx-4 and FSP1-CoQ_10_-NADH pathways in SIO-induced RPE degeneration pathogenesis. It has been shown that a higher level of iron was detected in post-mortem retinas with RPE degeneration when compared with controls [32]. Iron accumulation in the RPE also led to retinal degeneration with features of the RPE degeneration [29, 33]. Deferiprone, an iron chelator, is also shown to protect against retinal degeneration caused by light damage, sodium iodate, and the rd6 mutation [34]. Overall, our findings together with others suggest that imbalanced iron homeostasis and subsequent development of ferroptosis play a novel role in RPE degeneration (Fig. 8).

RCD, including necroptosis, pyroptosis, and ferroptosis in RPE cells has been shown to be involved in RPE degeneration pathogenesis [14, 20, 30]. Necroptosis in RPE cells was demonstrated in SIO-induced cell death and retinal damage [14]. The involvement of NLRP3-mediated pyroptosis in RPE cell death was identified in amyloid β oligomers-induced RPE degeneration models [30]. Besides, ferroptosis was shown in SIO-induced ARPE-19 cell damage in a recent study [35]. However, this study focused on the classical pathway of ferroptosis without a loss-of-function study such as GPx-4 knockdown. The authors mainly showed the protective effect of 24-h Fer-1 pre-treatment at 100 μM on ARPE-19 cells (an RPE cell line) upon SIO-induced damage. In contrast, we performed ferroptosis studies on primary HRPEpiC, ARPE-19 cell line, and mice using both pharmacological and loss-of-function strategies. We observed similar Fer-1 protective results on APRE-19 cells that were associated with the classical pathway of ferroptosis (GSH-GPx-4), but at a lower dosage after a shorter pre-treatment period. Fer-1 is a bioactive small molecule (molecular weight 262.35) classified as an antioxidant and cytoprotectant. It was identified as the first ferroptosis inhibitor and has been used in numerous in vitro and in vivo studies based on its efficacy in anti-lipid peroxidation in ferroptosis [36–40]. More importantly, we found that Fer-1 pre-treatment at 50 μM for 3 h effectively protected SIO-induced primary HRPEpiC cell death by decreasing MDA level. Based on these findings, we also discovered dramatic changes in subcellular level and in glutathione metabolisms when both types of RPE cells were exposed to SIO. We further investigated the role of the novel pathway of ferroptosis (FSP1-CoQ_10_-NADH) in SIO-induced RPE degeneration. Finally, we confirmed this phenomenon in vivo with a retinal function test ERG.

DFO is an FDA-approved iron-chelating agent for the treatment of acute iron intoxication and chronic iron overload. It chelates mostly Fe^3+^. It is also a representative inhibitor of ferroptosis and has been used extensively for various disease models [40]. However, due to DFO’s toxicity to the retina probably by inducing iron deficiency in the RPE, we did not perform in vivo study using DFO [41].

One of the limitations of this study is that the in vivo use of Fer-1 may not be recommended [16] due to the possible stability issues and short plasma half-life [42]. Fer-1 is a bioactive small molecule (molecular weight 262.35) classified as an antioxidant and cytoprotectant. It was identified as the first ferroptosis inhibitor and has been used in several in vitro and in vivo studies based on its efficacy in anti-lipid peroxidation in ferroptosis [36–40]. In our in vivo study, we chose a dosage of Fer-1 (2 mg/kg/day) that has been used in other in vivo studies [43–45]. We also performed daily administration of Fer-1, hoping that this may compensate for its short plasma half-life. In fact, we confirmed the protective effect of Fer-1 on RPE degeneration. As there was another report using Fer-1 in mice [46], we hoped that the use of the experimental animals in our study was well justified. Indeed, our animal data provided important in vivo evidence on the role of ferroptosis in RPE degeneration. Our in vivo results discovered a strong protective effect of Fer-1 on SIO-induced RPE degeneration, with significantly improved retinal thickness and function according to H&E staining results and ERG data. We next analyzed the involvement of the classical ferroptosis pathway in mouse RPE and found remarkable iron overload and impaired glutathione metabolism with increased lipid peroxidation. Based on these discoveries, we further investigated the association of a novel ferroptosis signaling and found that SIO-induced significant activation of the FSP1-CoQ_10_-NADH pathway in the RPE, which was reversed by Fer-1 treatment. We believe that these findings will contribute to the understanding of RPE degeneration pathology and to the development of new pharmacological agents. GPX4 and FSP1 are two independent pathways in ferroptosis in a variety of cell types [18]. Whether GPX4 or FSP1 overexpression could rescue iFSP1 or GPX4 siRNA phenotypes in RPE cells is also a way to validate the relationship of the two pathways in the future study.

In conclusion, our in vitro and in vivo studies demonstrated for the first time the involvement of the FSP1-CoQ_10_-NADH pathway in SIO-induced RPE degeneration, in addition to the classical GPx-4-GSH pathway. Inhibition or promotion of ferroptosis could rescue or aggravate RPE degeneration accordingly. This study provides important information for developing novel RPE degeneration therapies by targeting ferroptosis in the regulation of iron imbalance and lipid peroxidation.

## Materials and methods

### Cell culture and chemical treatment

Primary human retinal pigment epithelial cell (HRPEpiC) (Catalog#6540, Homp sapiens, ScienCell) and human adult retinal pigment epithelial cell line 19 (ARPE-19) (Homo sapiens, ATCC^®^ CRL2302™) were obtained from ATCC. Primary HRPEpiC cells within three passages were cultured in T25 flasks coated with Poly-L-lysine with DMEM/F-12 (Dulbecco’s Modified Eagle Medium/Nutrient Mixture F-12) (12400-016, Gibco, Thermo Fisher Scientific, Waltham, Massachusetts, USA) medium containing 1% epithelial cell growth supplement (EpiCGS), 10% fetal bovine serum (FBS) and 1% penicillin/streptomycin (P/S). ARPE-19 cell culture work was performed as previously described [30]. Briefly, cells within ten passages were cultured in Dulbecco’s Modified Eagle Medium/Nutrient Mixture F-12 (12400-016, Gibco, Thermo Fisher Scientific, Waltham, Massachusetts, USA) supplemented with FBS (10%) and 1% P/Sin 5% CO_2_ at 37 °C.

For pre-treatment experiments, cells were seeded in a 12-well plate (5 × 10^4^ cells/well) (665 180, Greiner-Bio, Kremsmünster, Austria) pre-treated with Fer-1 (0, 10, 25, 50, 100 μM, SML0583, Sigma Aldrich, Missouri, USA) or DFO (0, 5, 15, 25, 50 μM, D9533, Sigma Aldrich, Missouri, USA) for 3 h before subjecting them to sodium iodate (SIO, S4007, Sigma Aldrich, Missouri, USA) challenge for 24 h and harvested for analyses. The same concentration of DMSO and 1×PBS were used for the controls of Fer-1 and DFO, respectively. Ferroptosis suppressor protein 1 inhibitor at different concentrations (0, 1, 2, 5, 10 μM) (150651-39-1, MedChemExpress, Monmouth Junction, USA) were added to ARPE-19 cell culture medium for 24 h with or without subsequent treatment of SIO for 24 h.

### Assessment of cell viability

Cell viability was evaluated by CCK-8 assay as reported [30]. Cells were seeded in 96-well plate (5 × 10^3^ cells/well) (655 180, Greiner-Bio, Kremsmünster, Austria) pre-treated with Fer-1 (0, 10, 25, 50, 100 μM) or DFO (0, 5, 15, 25, 50 μM) for 3 h. Bright field images were obtained for assessment of morphological changes by TE2000 inverted microscopic imaging system (Nikon, Tokyo, Japan). iFSP1, an FSP1 inhibitor was administrated to the ARPE-19 cells at different concentrations (μM) with or without SIO (20 mM) treatment. iFSP1 stock was dissolved in DMSO while SIO was dissolved in PBS. For the control normalization experiment, the following experimental groups were included: (1) PBS + DMSO; (2) PBS + iFSP1; (3) SIO + iFSP1; (4) SIO + DMSO. The DMSO concentration present in the highest dosage of iFSP1 used (10 μM) was 0.1%, which had no cytotoxic effect. The DMSO concentrations present in each dosage of iFSP1 (0, 1, 2, 5, 10 μM) corresponded to 0%, 0.01%, 0.02%, 0.05%, 0.1%, respectively. In addition, PBS + iFSP1 + Fer-1 and SIO + iFSP1 + Fer-1 groups were supplemented to test if iFSP1 induces ferroptosis and not another kinds of cell death. ARPE-19 cells were pre-treated with 50 μM Fer-1 for 3 h before subjecting them to iFSP1 (0, 1, 2, 5, 10 μM or SIO (20 mM) for 24 h respectively, followed by the measurement of cell viability. Cell viability was also illustrated using LIVE/DEAD Cell Imaging Kit (488/570) (R37601, Thermo Fisher Scientific, Waltham, Massachusetts, USA) according to the manufacturer’s instructions [47]. The cells were imaged by TE2000 inverted microscopic imaging system (Nikon, Tokyo, Japan).

### GPx-4 small interfering RNA and transfection

Small interfering RNA (siRNA) targeting at GPx-4 (AM16708, Thermo Fisher Scientific, Waltham, Massachusetts, USA) and negative control siRNA (AM4611, Thermo Fisher Scientific, Waltham, Massachusetts, USA) were transfected into ARPE-19 cells using Lipofectamine 3000 (L3000001, Thermo Fisher Scientific, Waltham, Massachusetts, USA) and Opti-MEM™ I Reduced Serum Medium (31985070, Thermo Fisher Scientific, Waltham, Massachusetts, USA) according to manufacturer’s instruction. Transfected cells were then cultured for 48–72 h at 37 °C and harvested for analysis. Transfection efficacy was evaluated by Western blotting to assess the expression of GPx-4.

### Animals

In total, 6–8-week-old wildtype C57BL/6J male mice were obtained from a specific pathogen-free level animal facility at Centre for Comparative Medicine Research, The University of Hong Kong with 12 h light/dark cycle. The experimental procedures were approved by the Committee on the Use of Live Animals in Teaching and Research of The University of Hong Kong. For the establishment of RPE degeneration in vivo, mice received a single intraperitoneal injection of sodium iodate (SIO, S4007, Sigma Aldrich, Missouri, USA) dissolved in PBS at 30 mg/kg/day and 50 mg/kg/day. The extent of RPE damage was assessed and the RPE degeneration phenotype was successfully induced 3 days after a single intraperitoneal injection of SIO at 30 mg/kg. Therefore, in subsequent studies, mice were randomly divided into three groups: control group, SIO group, and Fer-1 group. Mice in the SIO group were given one SIO injection at 30 mg/kg (*n* = 6) on day 3. The optimized phenotype of RPE degeneration was confirmed 3 days after SIO injection. Therefore, mice in the Fer-1 group were treated daily with an intraperitoneal injection of Fer-1 (2 mg/kg/day in 2% DMSO in PBS, *n* = 6) starting at this time (Fig. 3A). Vehicle-treated animals received an equal volume of 2% DMSO in PBS (*n* = 6). On day 14, mice were sacrificed by pentobarbital overdose.

Mouse survival rate was estimated as follows. Survival rate on day *N* = 100% × (Total number of mice at the beginning of the experiment − dead number of mice from day 0 of the experiment)/Total number of mice at the beginning of the experiment. Body weight was monitored by daily weighing using a balance (PB-1200, Oriental Scales, Hong Kong). Daily IOP was measured by a portable rebound tonometer (Tonolab; Icare, Espoo, Finland) according to the manufacturer’s instruction. Briefly, the tonometer was handheld 1–4 mm in front of the mouse’s central cornea without anesthesia. A value was generated after automatic averaging of 6 consecutive tests by the tonometer. Three values of each eye were collected and averaged on day 14.

For animal sample collection and processing, mouse RPE/choroid was isolated from enucleated eyeballs as described previously [48]. In brief, after removal of the neural retina, the RPE/choroid was immersed in 200 μl protein lysis buffer for 1 h on ice with frequent gentle tapping to break the brownish RPE clumps. It was subsequently transferred to a new centrifuge tube and kept on ice for 30 min to release the RPE protein.

### Electroretinogram

ERG is used to test electrical activity and responsiveness of the retina upon light stimulation [22]. Waveforms recorded are separated into two components: a-wave and the subsequent b-wave. The a-wave is generated by photoreceptors while b-wave reflects the response from bipolar cells and other layers of the outer retina. ERG was therefore measured as reported [23–26] to reflect retinal function. Briefly, mice were dark-adapted for at least 12 h and anesthetized with ketamine (70 mg/kg, i.p.) and xylazine (7 mg/kg, i.p.). Pupils were dilated with 1% tropicamide (1% Mydriacyl, Alcon, Alcon-Couvreur, Belgium) and the cornea was anesthetized with 0.5% Alcaine (0.5 % proparacaine hydrochloride, Alcon, Alcon-Couvreur, Belgium). ERG was recorded (Diagnosys, Bengaluru, India) using a gold contact lens electrode, a reference electrode, and a ground electrode at two intensities (3 cd s/m^2^ and 10 cd s/m^2^). An ERG software (Epsion V5) was used to analyze the waveforms.

### Measurement of the iron level

Intracellular iron levels in human primary HRPEpiC, ARPE-19 cells, and mouse RPE were measured by iron assay kit (ab83366, abcam, Cambridge, United Kingdom) as described previously [49]. In brief, proteins were harvested from cultured cells and mouse retina. After estimation of protein concentration, samples and iron standards were incubated with working reagents in a 96-well plate for 60 min at 37 °C. Absorbance at 593 nm was measured with a microplate reader (ELx800, BioTek, Hong Kong, China). The iron concentration of each sample was normalized with protein concentration.

### Assessment of malondialdehyde (MDA) level

Intracellular MDA levels in human primary HRPEpiC, ARPE-19 cells, and mouse RPE were measured with an MDA detection kit (S0131S, Beyotime Biotechnology, Shanghai, China) following the manufacturer’s instructions. MDA concentration in the samples was determined according to the standard curve and normalized with the corresponding protein concentrations.

### Detection of GSH + GSSG/GSH level

GSH + GSSG/GSH levels in human primary HRPEpiC, ARPE-19 cells, and mouse RPE were detected by GSH + GSSG/GSH Assay Kit (Colorimetric) (ab239709, abcam, Cambridge, United Kingdom) as described previously [50]. Total GSH, including GSH and GSSG was measured using GSH reductase to reduce GSSG in the samples, which subsequently was oxidized by 5,5′-dithio-bis (2-nitrobenzoic acid) (DTNB), thereby producing a yellowish product, 5′-thio-2-nitrobenzoic acid (TNB). Total GSH was determined by measuring the absorbance with a microplate reader (ELx800, BioTek, Hong Kong, China) at 412 nm. GSSG was determined by removing endogenous GSH with GSH scavenger, followed by oxidation of DTNB. The level of GSH was calculated by subtracting the content of GSSG from the total GSH. Sample protein concentrations measured were used for normalization of GSH + GSSG/GSH level.

### Plasmid preparation

The plasmids, pCDH-CMV-AIFM2-3Flag-tRFP-F2A-Neo and pCDH-CMV-MCS-SBP-tRFP-F2A-Neo-3Flag were obtained from Hanyi biology limited, Guangzhou, China. Both plasmids were firstly transformed into competent cells (18265017, Thermo Fisher Scientific, Waltham, Massachusetts, USA). The presence of RFP signal indicated a successful transfection of cells by plasmids carrying both RFP and FSP1 DNA. They were thawed on ice and placed in two chilled polypropylene tubes in 50 µl aliquots. In total, 1 µl of each plasmid was immersed in the 50 µl cells, added to competent cells and incubated for 30–40 min on ice, followed by heat shock at 42 °C for 2 min and then immediately chilled in ice for 1–2 min. The competent cells and plasmid mixture were added into 500 µl Super Optimal Broth (S.O.C) medium with constant shaking at 37 °C overnight. In total, 10 µl or 50 µl of the mixture was spread on a lysogeny broth plate supplemented with 100 µg/ml ampicillin. All plates were cultured upside down overnight at 37 °C. On the next day, lysogeny broth plates were examined. Single colonies were isolated and incubated in tubes containing 15 ml lysogeny broth with 100 µg/ml ampicillin with constant shaking at 37 °C overnight. The turbid tubes were then centrifuged at 4000 rpm for 10–15 min, followed by plasmid extraction using Purelink Quick Plasmid Miniprep kit (REF K210010, Invitrogen, Waltham, Massachusetts, USA).

### NAD/NADH assay

NAD/NADH levels in ARPE-19 cells and mouse retina were detected using NAD/NADH assay kit (Colorimetric) (ab65348, abcam, Cambridge, United Kingdom) as described previously [51]. Cells or tissues were washed with cold PBS and harvested from 6-well plate. Cells or tissues were homogenized in NADH/NAD extraction buffer, followed with assay procedures according to kit instructions. Levels of NADH/NAD were calculated based on the standard curve.

### The enzyme-linked immunosorbent assay (ELISA)

RPE cells were harvested and incubated with protein lysis buffer. After two free-thaw cycles, the homogenates were centrifuged, and the supernatant was assayed. Intracellular CoQ_10_ (ubiquinone) was determined using CoQ_10_ ELISA kit (EKC33185, Biomatik, Ontario, Canada) following the manufacturer’s instructions.

### Retinal pigment epithelium/choroid flatmounts

C57BL/6J mice were sacrificed with pentobarbital overdose, and eyeballs were enucleated. The lens and the neuroretina were removed carefully. The RPE/choroid was flat-mounted on the glass slide, and examined by immunohistochemistry assays.

### Immunohistochemistry

Cultured cells were fixed in 4% paraformaldehyde for 1 h and blocked with 5% goat serum with 0.5% Triton X-100. Eyeballs were collected and fixed in 4% paraformaldehyde in PBS for 1 h and embedded in paraffin. Five-µm thick cross-sections with the optic nerve stump were blocked with 5% goat serum with 0.5% Triton X-100 for 1 h. Subsequently, cells and mouse retinal sections were incubated overnight at 4 °C with anti-4-Hydroxynonenal antibody (1:1000, MA5-27570, Invitrogen, Waltham, Massachusetts, USA) and anti-AMID antibody (B-6) (1:1000, sc-377120, Santa Cruz, CA, USA), followed by goat anti-mouse IgG H&L (Alexa Fluor® 488, 1:500, ab150113, Cambridge, United Kingdom). DAPI (1:1000, ab228549, abcam, Cambridge, United Kingdom) was used to stain the nuclei. A TE2000 inverted microscopic imaging system (Nikon, Tokyo, Japan) was used to acquire fluorescence images. Five images were taken for each sample in all experimental groups, with duplicates in independent experiments. Image J was used to analyze the immunofluorescence intensity of all images obtained from each group after background subtraction. Representative images were presented.

### Transmission electron microscope (TEM)

Cell morphology and mitochondria changes were examined by TEM. ARPE-19 cells with/without SIO challenge were trypsinized, washed with 1xPBS, and centrifuged. The cell pellets were resuspended in 4% paraformaldehyde, followed by epoxy resin embedding and staining with uranyl acetate and lead citrate. Samples were examined with a CM-100 TEM (Philips, Amsterdam, the Netherlands).

### Western blotting

An equal amount of proteins was loaded into the polyacrylamide gel (12.5%) and transferred to polyvinylidene difluoride membranes. After blocking with 5% non-fat milk and 0.001% Tween-20 in Tris-buffered saline (1×TBST) for 1 h, the membrane was first incubated with anti-GPx-4 antibody (1:1000, sc-166120, Santa Cruz, CA, USA), anti-ACSL4 antibody (1:1000, sc-365230, Santa Cruz, CA, USA) and anti-AMID antibody (B-6) (1:1000, sc-377120, Santa Cruz, CA, USA) at 4 °C overnight followed by incubation with rabbit anti-mouse IgG H&L (Alexa Fluor^®^ 488) (1:500, ab150117, abcam, Cambridge, United Kingdom) for 1 h at room temperature. Band visualization was performed by Amersham Imager 680 imager, and the intensity was analyzed by Image J.

### Statistics

Experimental results were presented as mean ± standard error of the mean. Student’s *t* tests or one-way ANOVA followed by LSD test were used for data analysis using SPSS 20.0 software (SPSS Inc., Chicago, USA). *p* < 0.05 was considered statistically significant.

### Reporting summary

Further information on research design is available in the Nature Research Reporting Summary linked to this article.

### Supplementary information

Figure S1

Figure S2

Figure S3

Figure S4

Supplementary figure legends

Reporting Summary

Supplementary material

List of materials

## Data Availability

The data that support the findings of this study are available from the corresponding author upon reasonable request.
