# Correction: Involvement of FSP1-CoQ_10_-NADH and GSH-GPx-4 pathways in retinal pigment epithelium ferroptosis

**DOI:** 10.1038/s41419-024-06555-3

**Published:** 2024-03-08

**Authors:** Ming Yang, Michelle Grace Tsui, Jessica Kwan Wun Tsang, Rajesh Kumar Goit, Kwok-Ming Yao, Kwok-Fai So, Wai-Ching Lam, Amy Cheuk Yin Lo

**Affiliations:** 1 Department of Ophthalmology, Li Ka Shing Faculty of Medicine, The University of Hong Kong, Hong Kong, China; 2 School of Biomedical Sciences, Li Ka Shing Faculty of Medicine, The University of Hong Kong, Hong Kong, China; 3 State Key Laboratory of Brain and Cognitive Sciences, The University of Hong Kong, Hong Kong, China; 4 GHM Institute of CNS Regeneration, Jinan University, Guangzhou, China

**Keywords:** Apoptosis, Neurodegenerative diseases, Experimental models of disease

Correction to: *Cell Death and Disease* 10.1038/s41419-022-04924-4, published online 18 May 2022

In the original version of this article, the images in the “1mM” and “5mM” panels in Figure 1c were mistakenly used. The error is now rectified and the correct Figure 1c is shown below. The authors sincerely apologize to the editors and readers for any confusion or inconvenience this mistake may have caused.
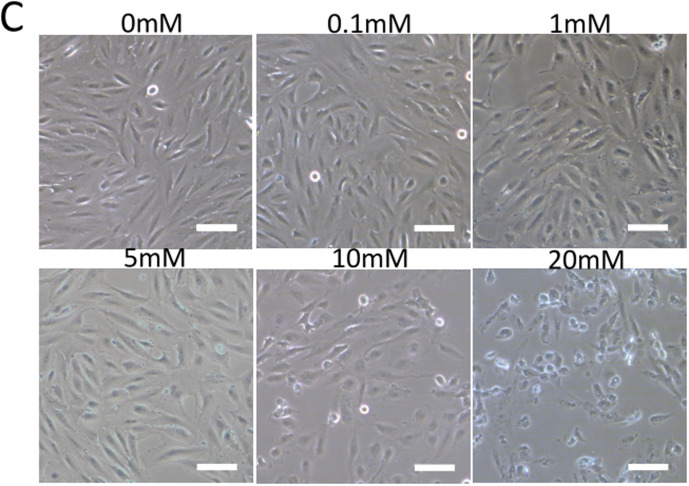

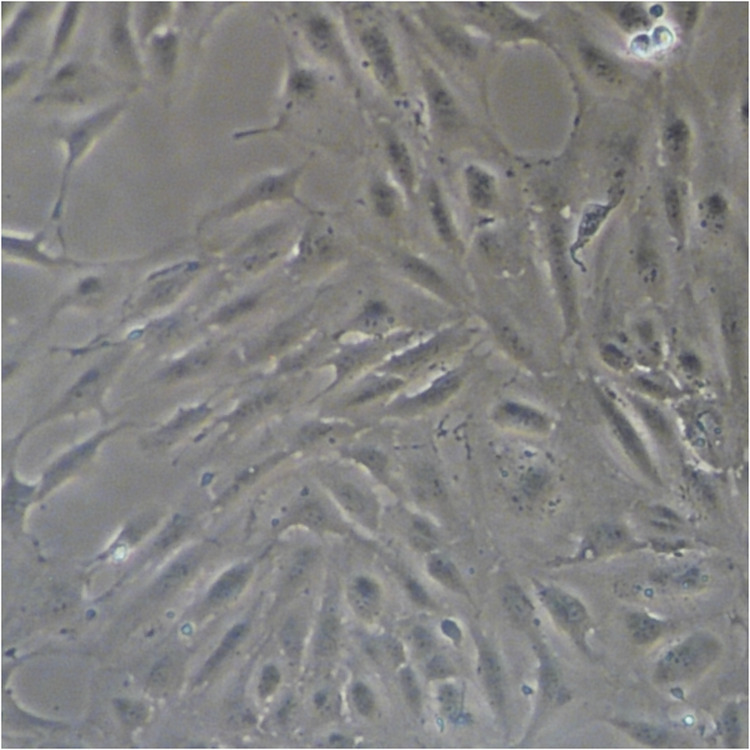

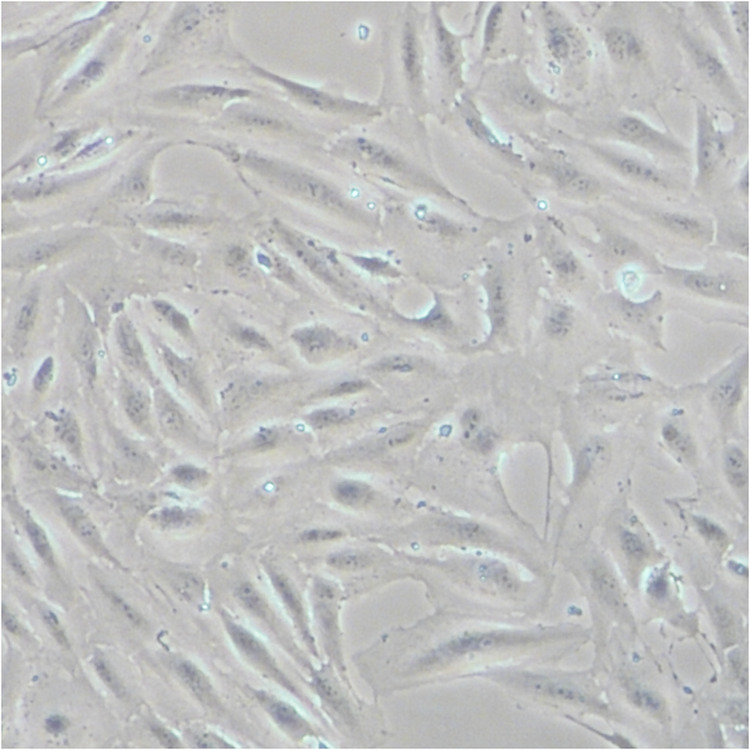


The original article has been corrected.

