# Supplementary figures and images for: Involvement of FSP1-CoQ10-NADH and GSH-GPx-4 pathways in retinal pigment epithelium ferroptosis
Source: Cell Death Dis. 2022 May 18;13(5):468. doi: 10.1038/s41419-022-04924-4 (PMC9117320; doi:10.1038/s41419-022-04924-4)

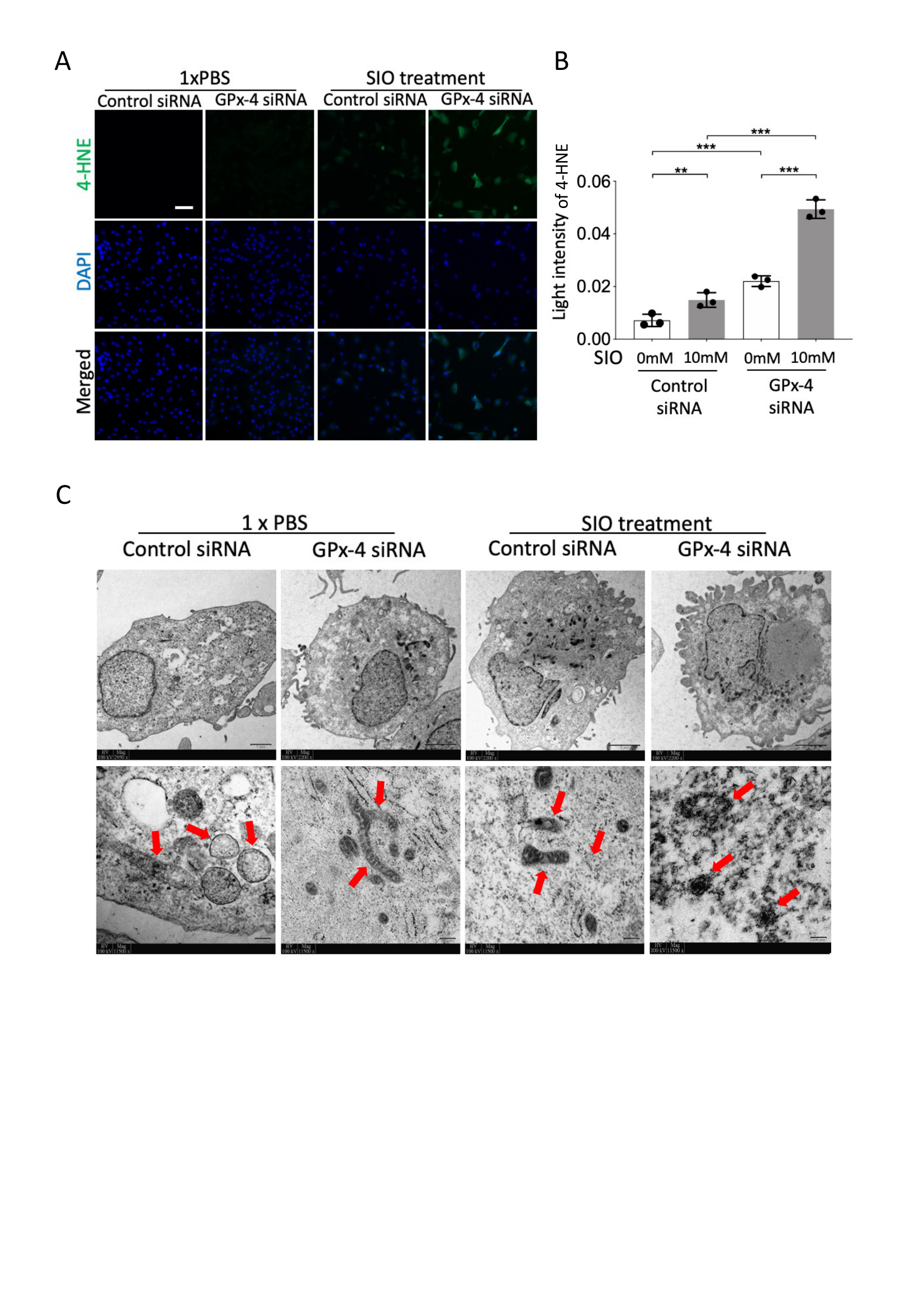

Supplement: Supplementary file 1 — Figure S1 [file 41419_2022_4924_MOESM1_ESM.tif]

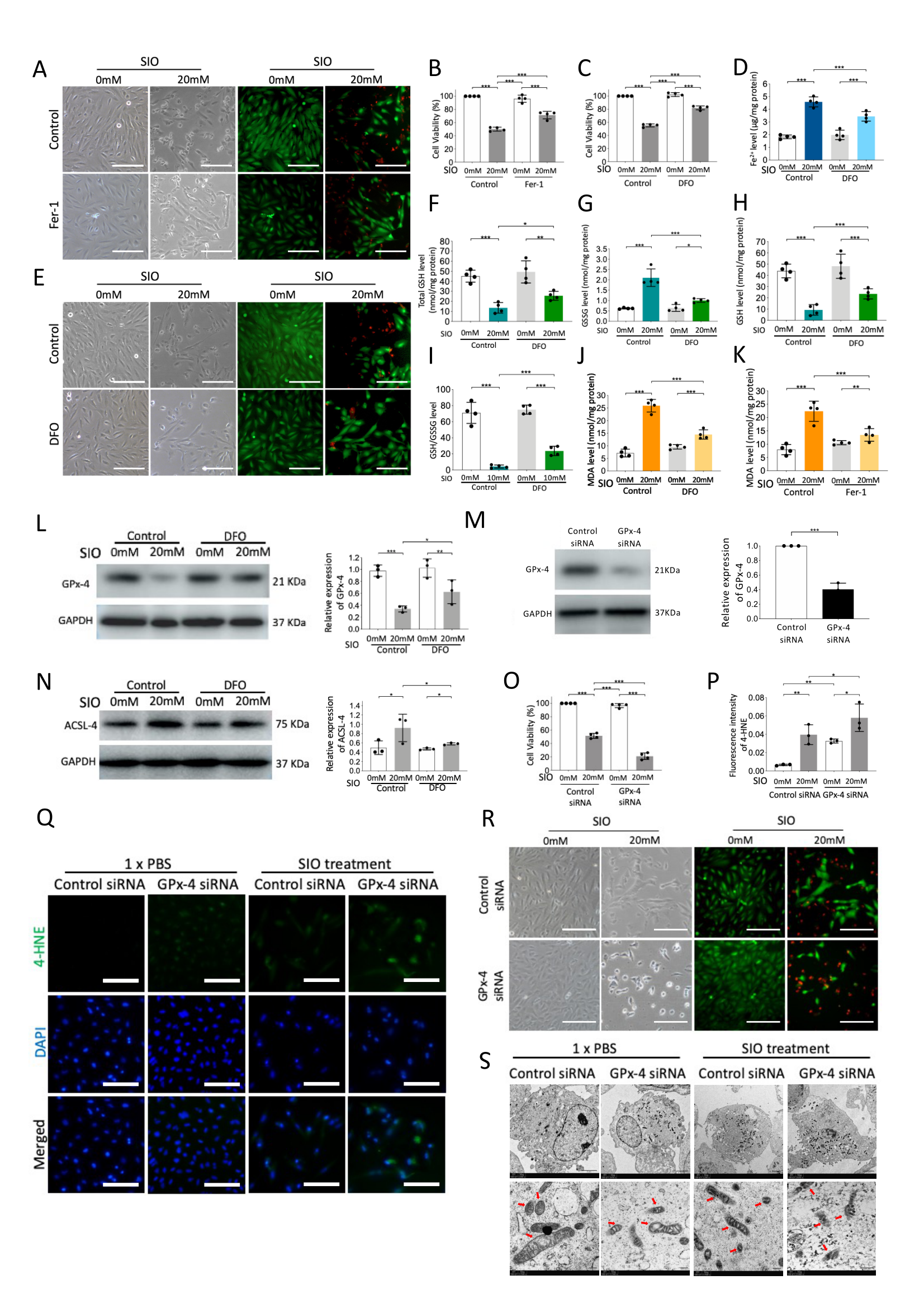

Supplement: Supplementary file 2 — Figure S2 [file 41419_2022_4924_MOESM2_ESM.tif]

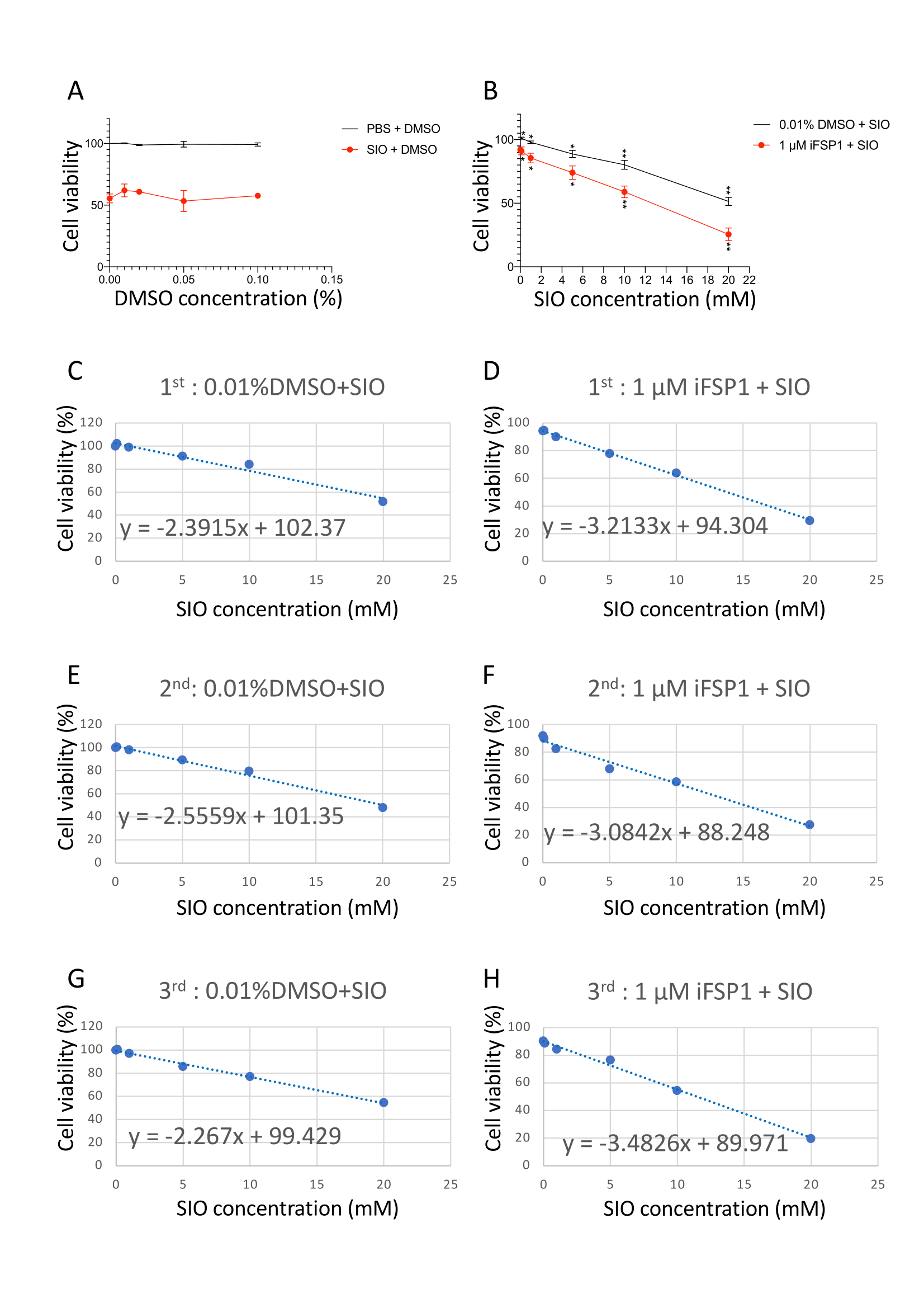

Supplement: Supplementary file 3 — Figure S3 [file 41419_2022_4924_MOESM3_ESM.tif]

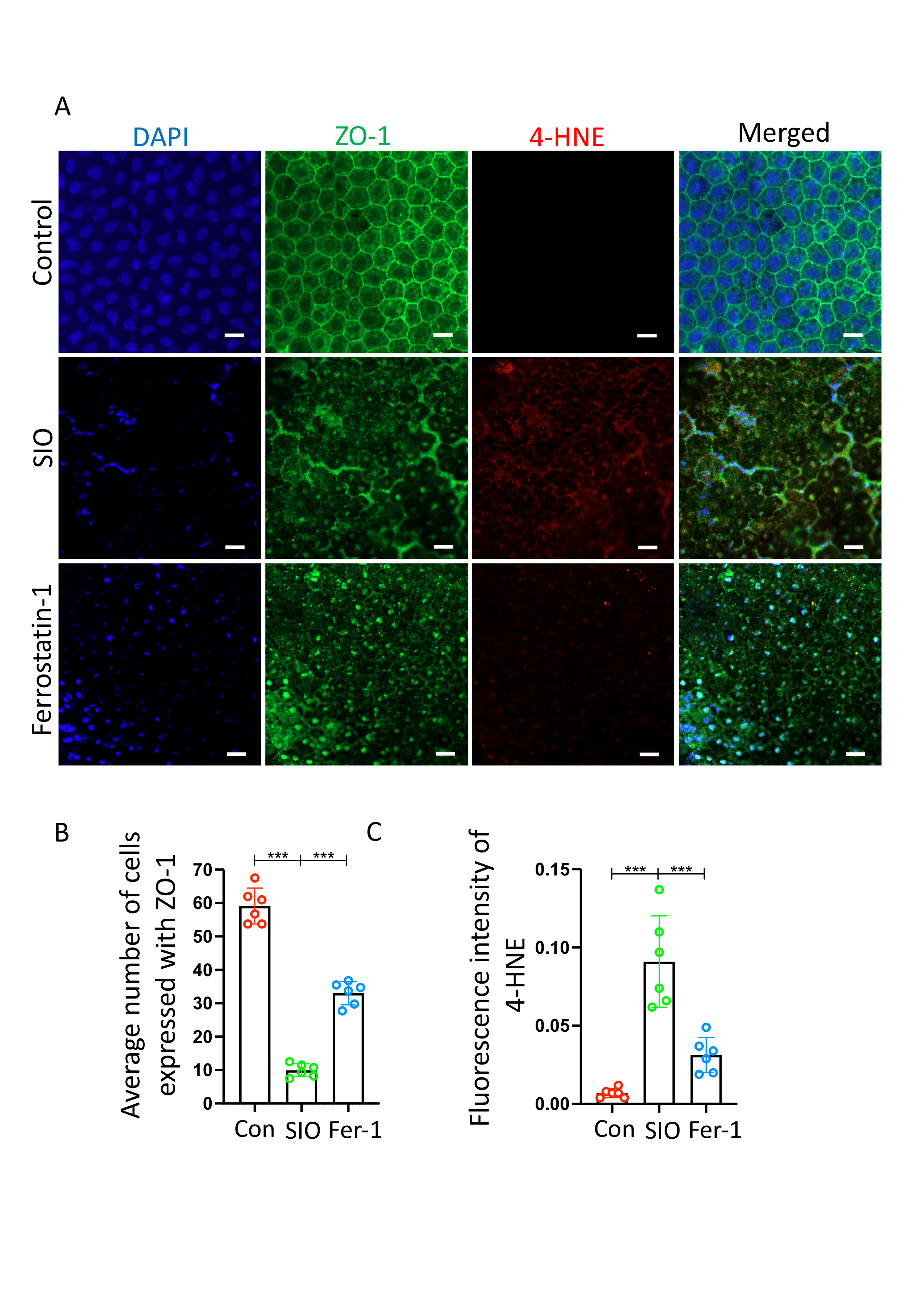

Supplement: Supplementary file 4 — Figure S4 [file 41419_2022_4924_MOESM4_ESM.tif]
