# Supplementary material for: Involvement of FSP1-CoQ10-NADH and GSH-GPx-4 pathways in retinal pigment epithelium ferroptosis
Source: Cell Death Dis. 2022 May 18;13(5):468. doi: 10.1038/s41419-022-04924-4 (PMC9117320; doi:10.1038/s41419-022-04924-4)
Supplement: Supplementary file 5 — Supplementary figure legends [file 41419_2022_4924_MOESM5_ESM.docx]

**Supplementary figure legends**

Figure S1. Ferroptosis regulates SIO-induced human primary HRPEpiC cell death.

A. 4-HNE immunofluorescence in human primary HRPEpiC cell, SIO and/or GPx-4 siRNA. B. Quantitative analysis of the fluorescence intensity of 4-HNE. Scale bar = 50 µm. n = 3 (independent experiments). C. Transmission electron microscopy images of human primary HRPEpiC cell with/without GPx-4 knockdown after SIO challenge. Scale bar (the first row) = 2 µm. Scale bar (the second row) = 200 nm. Red arrows: mitochondria. *p<0.05, **p<0.01, ***p<0.001

Figure S2. Ferroptosis regulates SIO-induced ARPE-19 cell death.

Fer-1 (50 µM) or its vehicle (0.14% DMSO) was given to ARPE-19 cells 3 hours before control (PBS) or SIO (20 mM) treatment. DFO (25 µM) or its vehicle (PBS) was given to ARPE-19 cells 3 hours before control (PBS) or SIO (20 mM) treatment. n= 3 or 4 (independent experiments). A. Phase-contrast and fluorescence microscope images of cell morphology and Live/dead cell staining of SIO-exposed ARPE-19 cells with/without Fer-1 pre-treatment are shown. Scale bar = 100 µm. B. CCK-8 cell viability assay results of the effect of Fer-1 on 20 mM SIO-induced ARPE-19 cell death. C. CCK-8 cell viability assay results of the effect of DFO on 20 mM SIO-induced ARPE-19 cell death. D. Labile iron levels in SIO-exposed ARPE-19 cells with/without DFO pre-treatment. E. Phase-contrast and fluorescence microscope images of cell morphology and Live/dead cell staining of SIO-exposed ARPE-19 cells with/without DFO pre-treatment are shown. Scale bar = 100 µm. F. Total GSH level of SIO-exposed ARPE-19 cells with/without DFO pre-treatment. G. GSSG level of SIO-exposed ARPE-19 cells with/without DFO pre-treatment. H. GSH level of SIO-exposed ARPE-19 cells with/without DFO pre-treatment. I. GSH/GSSG level of SIO-exposed ARPE-19 cells with/without DFO pre-treatment. J. MDA level of SIO-exposed ARPE-19 cells with/without DFO pre-treatment. K. MDA level of SIO-exposed ARPE-19 cells with/without Fer-1 pre-treatment. L. The expression of GPx-4 of SIO-exposed ARPE-19 cells with/without DFO pre-treatment showed by Western blot. M. The expression of GPx-4 with/without knock-down. N. The expression of ACSL-4 of SIO-exposed ARPE-19 cells with/without DFO pre-treatment showed by Western blot. O. CCK-8 cell viability assay results of the effect of GPx-4 knockdown on 20 mM SIO-induced ARPE-19 cell death. Q. 4-HNE immunofluorescence after GPx-4 knockdown in 20 mM SIO-induced ARPE-19 cell death. Scale bar = 50 µm. R. Phase-contrast and fluorescence microscope images of cell morphology and Live/dead cell staining of ARPE-19 cells with/without GPx-4 knockdown after SIO challenge. Scale bar = 100 µm. S. Transmission electron microscopy images of ARPE-19 cells with/without GPx-4 knockdown after SIO challenge. Scale bar (the first row) = 2 µm. Scale bar (the second row) = 200 nm. Red arrows: mitochondria. *p<0.05, **p<0.01, ***p<0.001.

Figure S3. Effect of iFSP1 on SIO dose-response in ARPE-19 cells.

A. Cell viabilities of iFSP1 control and SIO control (control normalization for figure 5A). iFSP1 was dissolved in DMSO, and SIO was dissolved in PBS. Four experimental groups were designed: 1. PBS + DMSO; 2. SIO + DMSO; 3. SIO + iFSP1; 4. PBS + iFSP1. B. The effect of low concentration of iFSP1 (1 µM) on cell viability of SIO-exposed ARPE-19 cells. n = 3 independent experiments. C. Dose-response of ARPE-19 cells to increasing concentration of SIO in the presence of 0.01% DMSO (1^st^ experiment, slope= -2.3915). D. Dose-response of ARPE-19 cells to increasing concentration of SIO in the presence of 1 µM iFSP1 (1^st^ experiment, slope= -3.2133). E. Dose-response of ARPE-19 cells to increasing concentration of SIO in the presence of 0.01% DMSO (2^nd^ experiment, slope= -2.5559). F. Dose-response of ARPE-19 cells to increasing concentration of SIO in the presence of 1 µM iFSP1 (2^nd^ experiment, slope= -3.0842). G. Dose-response of ARPE-19 cells to increasing concentration of SIO in the presence of 0.01%DMSO (3^rd^ experiment, slope= -2.267). H. Dose-response of ARPE-19 cells to increasing concentration of SIO in the presence of 1 µM iFSP1 (3^rd^ experiment, slope= -3.4826). *p<0.05, **p<0.01, ***p<0.001.

Figure S4. Ferroptosis regulates SIO-induced RPE degeneration in vivo (RPE flat-mount). A 4-HNE and ZO-1 immunofluorescence in mouse RPE flat-mount. B. Quantitative analysis of the average number of cells expressed with ZO-1. C. Quantitative analysis of the fluorescent intensity of 4-HNE Scale bar = 20 µm.
