# Supplementary material for: Involvement of FSP1-CoQ10-NADH and GSH-GPx-4 pathways in retinal pigment epithelium ferroptosis
Source: Cell Death Dis. 2022 May 18;13(5):468. doi: 10.1038/s41419-022-04924-4 (PMC9117320; doi:10.1038/s41419-022-04924-4)
Supplement: Supplementary file 8 — List of materials [file 41419_2022_4924_MOESM8_ESM.docx]

**List of materials**

| **Items** | **Classification** | **Source or reference** | **Identifiers** |
| --- | --- | --- | --- |
| **Animals** | C57BL/6J | Jackson Laboratories | 000664 |
| **Cells** | human primary HRPEpiC | Homp sapiens, ScienCell | Catalog#6540 |
| **Cells** | ARPE-19 | Homo sapiens, ATCC | CRL2302 ™ |
| **Chemical** | Ferrostatin-1 | Sigma Aldrich | Cat.-No. SML0583 |
| **Chemical** | Deferoxamine | Sigma Aldrich | Cat.-No. D9533 |
| **Chemical** | Sodium Iodate | Sigma Aldrich | S4007 |
| **Chemical** | Ferroptosis suppressor protein 1 inhibitor | MedChemExpress, | CAS No.: 150651-39-1 |
| **Kits** | Cell Counting Kit-8 | Greiner-Bio | Cat.-No. 655 180 |
| **Kits** | Live/Dead cell viability assay kit | ThermoFisher Scientific | L3224 |
| **Reagents** | Small interfering RNA (siRNA) targeting at GPx-4 (ThermoFisher Scientific,) | ThermoFisher Scientific | AM16708 |
| **Reagents** | Negative control siRNA | ThermoFisher Scientific | AM4611 |
| **Kits** | Iron assay kit | abcam | ab83366 |
| **Kits** | MDA detection kit | Beyotime Biotechnology | S0131S |
| **Kits** | GSH+GSSG/GSH detection kit | abcam | ab239709 |
| **Kits** | NAD/NADH assay kit (Colorimetric) | abcam | ab65348 |
| **Kits** | CoQ10 ELISA kit | Biomatik | EKC33185 |
| **Antibody** | 4-Hydroxynonenal | Invitrogen | MA5-27570 |
| **Antibody** | anti-AMID antibody (B-6) | Santa Cruz | sc-377120 |
| **Antibody** | (Alexa Fluor® 488) | abcam | ab150113 |
| **Software** | Graphpad prism7 | GraphPad Software |  |
| **Software** | Fiji/ImageJ | National Institute of Health |  |
| **Equipment** | LSM 880 confocal microscopy | Zeiss | LSM 880 |
| **Equipment** | Inverted microscopic imaging system | Nikon | TE2000 |
| **Equipment** | Transmission electron microscope (TEM) | Philips | CM-100 |
| **Equipment** | Electroretinogram (ERG) | Intelligent Hearing Systems |  |
